# ﻿First cytogenetic data on Afrotropical lutefishes (Citharinidae) in the light of karyotype evolution in Characiformes

**DOI:** 10.3897/compcytogen.v16.i2.79133

**Published:** 2022-06-21

**Authors:** Sergey A. Simanovsky, Dmitry A. Medvedev, Fekadu Tefera, Alexander S. Golubtsov

**Affiliations:** 1 Severtsov Institute of Ecology and Evolution, Russian Academy of Sciences, 33 Leninskij prosp., 119071 Moscow, Russia Severtsov Institute of Ecology and Evolution, Russian Academy of Sciences Moscow Russia; 2 National Fishery and Aquatic Life Research Center, Ethiopian Institute of Agricultural Research, P.O. Box 64, Sebeta, Ethiopia National Fishery and Aquatic Life Research Center, Ethiopian Institute of Agricultural Research Sebeta Ethiopia

**Keywords:** Africa, Characoidei, chromosomes, Citharinoidei, *Citharinus*, karyotype evolution

## Abstract

The Afrotropical lutefish family Citharinidae (Citharinoidei, Characiformes) comprises three genera with eight species in total. Although Citharinidae have been studied in terms of taxonomy and systematics, no cytogenetic information was available for any representative of the family. Furthermore, only one species out of 116 in Citharinoidei (*Distichodusaffinis* Günther, 1873) has been studied cytogenetically. Here, we report the karyotypes of *Citharinuscitharus* (Geoffroy St. Hilaire, 1809) from West Africa and *Citharinuslatus* Müller et Troschel, 1844 from Northeast Africa. The former has the diploid chromosome number 2n = 40 and the fundamental number FN = 80, while the latter has 2n = 44 and FN = 88. Hence, these karyotypes consist exclusively of bi-armed chromosomes. Such karyotypes were previously found in *D.affinis* and in many lineages of Neotropical species of another suborder of Characiformes, Characoidei. In contrast, the karyotypes dominated by uni-armed elements are typical for a number of phylogenetically basal lineages of Afrotropical and Neotropical Characoidei. We discuss the importance of our data on Citharinidae for the understanding of the karyotype evolution within the order Characiformes.

## ﻿Introduction

Characins, the order Characiformes, are classified into two suborders: Citharinoidei and Characoidei. The former includes two Afrotropical families: Citharinidae with eight species in three genera and Distichodontidae with 108 species in 16 genera, while the latter suborder (Characoidei) contains more than 2,000 species in two Afrotropical (Alestidae and Hepsetidae) and 20 Neotropical families (Nelson et al. 2016; Betancur et al. 2018; Froese and Pauly 2022). The suborders are undoubtedly monophyletic, while the monophyly of the order as a whole has been put into question by some studies of its molecular phylogeny (reviewed by Arcila et al. 2017; Betancur et al. 2018). The time of the divergence of the characin suborders is estimated between 115 and 145 Mya (Lavoué 2019, but also see Arroyave et al. 2013).

There is no cytogenetic information about any citharinid species, whereas the karyotype of the only distichodontid species, *Distichodusaffinis* Günther, 1873, was analyzed by Rab et al. (1998). In contrast, the extensive literature on the cytogenetics of Characoidei, both Afrotropical (Post 1965; Hinegardner and Rosen 1972; Krysanov and Golubtsov 2014; Carvalho et al. 2017; Mohamed et al. 2019) and Neotropical (reviewed by Oliveira et al. 1988, 2007, 2009; Galetti et al. 1994; Fenocchio et al. 2003; Nirchio et al. 2014; Pazian et al. 2018, Sassi et al. 2020), demonstrates the substantial variety of the karyotype structure and different modes of its evolution in hundreds of cytogenetically studied species.

Here, we present the first data on the karyotypes of two species of the genus *Citharinus* Cuvier, 1816. We then cytogenetically compare these species with the nearest studied relative, *D.affinis*, and other characins. Finally, we discuss the importance of these data for the understanding of the karyotype evolution within the order Characiformes.

## ﻿Materials and methods

Seven individuals of an undetermined sex (UD) of *Citharinuscitharus* (Geoffroy St. Hilaire, 1809), standard length (SL) of 61–91 mm, and three individuals (a female, a male and a UD individual) of *C.latus* Müller et Troschel, 1844, SL = 63–84 mm, were karyotyped. For each individual, at least 10 complete metaphases were analyzed to establish the diploid chromosome number and the karyotype structure. The total numbers of complete metaphase plates studied for each species were 101 and 42, respectively. *Citharinuscitharus* were purchased from the Nigerian aquarium fish dealers through the mediation of the company Aqua Logo Engineering (https://www.aqualogo-engineering.ru) in October of 2021, while *C.latus* individuals were collected in southwestern Ethiopia by the Joint Ethiopian-Russian Biological Expedition (JERBE) from the Alvero River just downstream of the Abobo Dam (7°52'23"N, 34°29'48"E) in November of 2017. This river belongs to the Sobat River drainage discharging into the White Nile in South Sudan. Nigerian fish were kept in the Moscow laboratory in a 100-l aquarium with permamently aerated and filtered water for one to ten days before treatment. Ethiopian fish were caught with a cast net and delivered in 80-l plastic containers into the field laboratory, where they were kept in permamently aerated water for several hours before treatment.

Before preparation, fish were treated intraperitoneally with 0.025% colchicine (0.01 ml / 1 g of their weight) for 1–2 hours (for *C.citharus*, in laboratory conditions) or 0.1% colchicine for 3–4 hours (for *C.latus*, in field conditions). Then, fish were euthanized with an overdose of tricaine methanesulfonate (MS-222), identified, measured with an accuracy of ±1 mm, dissected for gonad examination and tissue sampling, and preserved in 10% formaldehyde. Species identification was done based on the morphological characters (mostly, the number of scales in the lateral line for *C.citharus* and the relative size of adipose fin for *C.latus*, according to Gosse 1990; Golubtsov et al. 1995). Vouchers are deposited at the Severtsov Institute of Ecology and Evolution (Moscow), under provisional labels of JERBE.

Chromosome preparations were obtained from *C.citharus* following Bertollo et al. (2015) and from *C.latus* following Kligerman and Bloom (1977), with some modifications for both protocols. Briefly, the kidneys of *C.citharus* were suspended in 10 ml of a 0.075M KCl hypotonic solution and incubated for 20 min at room temperature; then 1 ml of the freshly prepared 3:1 methanol : acetic acid fixative was added and the cell suspension was centrifuged for 5 minutes at 1000 rpm. Afterwards, the supernatant was discarded, 5 ml of the fixative were added, and the cell suspension was kept at 4 °C for 15–20 min. These procedures were repeated two more times. After the third centrifugation and the elimination of the supernatant, 0.5–1.0 ml of the fixative was added and the final cell suspension was left for storage at -20 °C. To prepare chromosome spreads, several small drops of the cell suspension were released onto various sections of a slide, previously maintained in distilled water at 4 °C, then the slides were transferred to a hot plate (45 °C) for drying. As for *C.latus*, the kidney tissue was incubated with a 0.075M KCl hypotonic solution for 20 min and fixed in three changes of the 3:1 methanol : acetic acid fixative. To prepare slides, the fixed tissue was incubated with the 50% glacial acetic acid, suspended, and dropped onto hot slides (45 °C). The chromosome spreads of both species were stained conventionally with 4% Giemsa solution in a phosphate buffer solution at pH 6.8 for 8 min.

The chromosome spreads were analysed using an Axioplan 2 Imaging microscope (Carl Zeiss, Germany) equipped with a CV-M4^+^CL camera (JAI, Japan) and the Ikaros software (MetaSystems, Germany). Final images were processed using the Photoshop software (Adobe, USA). Karyotypes were established according to the centromere position following the nomenclature by Levan et al. (1964). Chromosomes were classified as metacentric (m) or submetacentric (sm), grouped according to their morphology and ordered by the decrease of their size. To determine the fundamental number (FN), metacentrics and submetacentrics were considered bi-armed.

## ﻿Results and discussion

**Figure 1.**
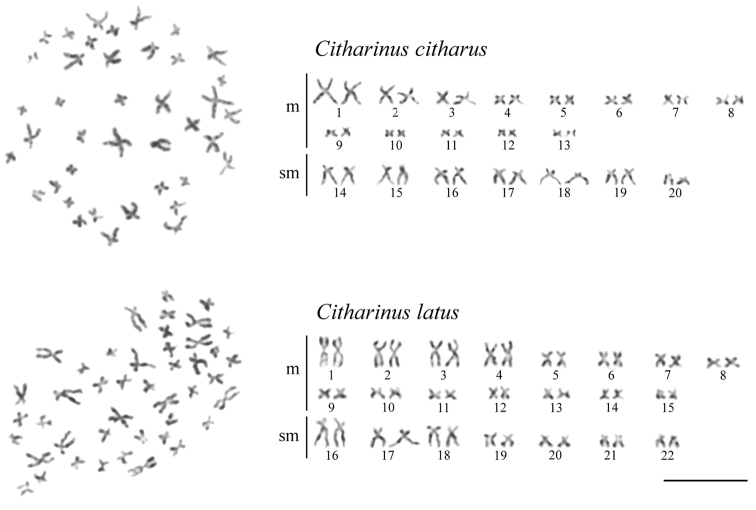
Metaphase chromosome plates (left) and karyotypes (right) of *Citharinuscitharus* and *C.latus* after conventional Giemsa staining. Scale bar: 10 μm.

The karyotype of *C.citharus* has 2n = 40 and consists of 26 metacentrics (m) and 14 submetacentrics (sm), the fundamental number FN = 80 (Fig. 1, above). The karyotype of *C.latus* has 2n = 44 and consists of 30 m and 14 sm, FN = 88 (Fig. 1, below). No distinguishable sex chromosomes were observed in complements of the two *Citharinus* species, similar to the report by Rab et al. (1998) for *Distichodusaffinis*.

*Citharinuscitharus* has nine chromosome pairs (nos. 1–3 and 14–19) noticeably larger than others, while *C.latus* has seven large chromosome pairs (nos. 1–4 and 16–18). This difference could be explained by two fusions of four pairs of smaller chromosomes (if the karyotype of *C.latus* is considered ancestral) or fissions of two pairs of larger chromosomes (if the karyotype of *C.citharus* is considered ancestral). However, another possible scenario would be an independent origin of karyotypes of the two *Citharinus* species. Namely, *D.affinis* exhibits 2n = 48 (Rab et al. 1998), while 2n = 50–54 is typical for the phylogenetically basal groups of Characoidei (Arai 2011; Machado et al. 2011; Cioffi et al. 2012; Krysanov and Golubtsov 2014; Arcila et al. 2017; Carvalho et al. 2017). Therefore, the two *Citharinus* karyotypes could evolve via the different numbers of chromosome fusions from an ancestral karyotype with the diploid chromosome number higher than those displayed by the two *Citharinus* species. Obviously, these scenarios are speculative due to the lack of cytogenetic data for the most of genera and species of Citharinoidei. However, our results, together with the basal location of the family Citharinidae in the phylogeny of all Citharinoidei and a distant location in this phylogeny of *D.affinis* (Arroyave et al. 2013; Betancur et al. 2018), suggest a substantial role of chromosome fusions/fissions in the evolution of Citharinoidei karyotypes.

Of note, all the three Citharinoidei species with studied karyotypes – both *Citharinus* species presented here and *D.affinis* studied by Rab et al. (1998) – have exclusively bi-armed chromosomes. However, due to the fragmentary cytogenetic data for Citharinoidei, we cannot reliably conclude whether this karyotype structure is typical for the suborder.

In comparison, the suborder Characoidei that is better studied cytogenetically demonstrates a wide variation in karyotype structures even in its basal groups. Specifically, karyotypes with exclusively bi-armed chromosomes are found in the family Crenuchidae (Oliveira et al. 2007; Arai 2011; Machado et al. 2011) which is a basal group of all Characoidei (Arcila et al. 2017). Of note, bi-armed karyotypes are also characteristic of some other families of the suborder, namely Anostomidae, Chilodontidae, Curimatidae and Prochilodontidae; however, they are nested far from the root of the Characoidei phylogeny (Arai 2011; Arcila et al. 2017). In contrast to Crenuchidae, two groups of other Characoidei families stem out rather close to the basal nodes but the karyotype structures of most of their members that were cytogenetically studied are enriched with or even dominated by uni-armed chromosomes. The first group consists of Neotropical Ctenoluciidae and Lebiasinidae, while the second group comprises Afrotropical Hepsetidae and Alestidae, as well as Neotropical Erythrinidae (Arai 2011; Cioffi et al. 2012; Krysanov and Golubtsov 2014; Arcila et al. 2017; Carvalho et al. 2017; Mohamed et al. 2019). The reported exceptions in these groups are genera *Hoplerythrinus* Gill, 1896 and *Hoplias* Gill, 1903 (Erythrinidae) whose karyotypes are almost completely dominated by bi-armed chromosomes (Arai 2011; Cioffi et al. 2012).

Importantly, our new data on the two Citharinoidei karyotypes suggest a revision of the current hypothesis about the ancestral chromosome number of the order Characiformes. Namely, based almost exclusively on the cytogenetic data from the other suborder of Characiformes, Characoidei, the chromosome number 2n = 54 was suggested to be ancestral for the whole order (Oliveira et al. 1998, 2007; Cioffi et al. 2012; Carvalho et al. 2017). However, cytogenetic data on the family Citharinidae and, in general, the suborder Citharinoidei is also important for the understanding of the karyotype evolution in Characiformes, because the family Citharinidae is a basal group for Citharinoidei, while Citharinoidei is a basal group for all Characiformes (Arcila et al. 2017). In this context, our data on Citharinidae suggest the possibility of a lower ancestral chromosome number for the order Characiformes and indicates the need of futher cytogenetic studies in the phylogenetically basal groups of Citharinoidei and Characoidei to clarify the evolution of the chromosome number and the karyotype structure in Characiformes.

On the other hand, some authors recently proposed a new hypothetical molecular phylogeny of the ray-finned fishes where the suborder Citharinoidei is separated into an order Cithariniformes and considered as a sister group to Characiformes + Siluriformes (Dornburg and Near 2021; Melo et al. 2021). Consequently, according to this new hypothesis, the karyotypes of Citharinoidei/Cithariniformes and Characoidei/Characiformes could evolve more independently. However, even in that case our new data, together with any future cytogenetic studies of Citharinoidei/Cithariniformes, will help to reconstruct the evolutionary history of karyotypes in a part of the superorder Ostariophysi.
